# *PaxA*, but not *PaxC*, is required for cnidocyte development in the sea anemone *Nematostella vectensis*

**DOI:** 10.1186/s13227-017-0077-7

**Published:** 2017-09-04

**Authors:** Leslie S. Babonis, Mark Q. Martindale

**Affiliations:** 1 Whitney Laboratory for Marine Bioscience, University of Florida, 9505 Ocean Shore Blvd, St. Augustine, FL 32080 USA; 2 Department of Biology, University of Florida, Gainesville, FL 32611 USA

**Keywords:** *PaxA*, *PaxC*, *Mef2*, *SoxB2*, Cell differentiation, Novelty, Gene regulatory network, Evolution, Nematocyte

## Abstract

**Background:**

*Pax* genes are a family of conserved transcription factors that regulate many aspects of developmental morphogenesis, notably the development of ectodermal sensory structures including eyes. *Nematostella vectensis*, the starlet sea anemone, has numerous *Pax* orthologs, many of which are expressed early during embryogenesis. The function of *Pax* genes in this eyeless cnidarian is unknown.

**Results:**

Here, we show that *PaxA*, but not *PaxC*, plays a critical role in the development of cnidocytes in *N. vectensis*. Knockdown of *PaxA* results in a loss of developing cnidocytes and downregulation of numerous cnidocyte-specific genes, including a variant of the transcription factor *Mef2*. We also demonstrate that the co-expression of *Mef2* in a subset of the *PaxA*-expressing cells is associated with the development with a second lineage of cnidocytes and show that knockdown of the neural progenitor gene *SoxB2* results in downregulation of expression of *PaxA*, *Mef2*, and several cnidocyte-specific genes. Because *PaxA* is not co-expressed with *SoxB2* at any time during cnidocyte development, we propose a simple model for cnidogenesis whereby a *SoxB2*-expressing progenitor cell population undergoes division to give rise to *PaxA*-expressing cnidocytes, some of which also express *Mef2*.

**Discussion:**

The role of *PaxA* in cnidocyte development among hydrozoans has not been studied, but the conserved role of *SoxB2* in regulating the fate of a progenitor cell that gives rise to neurons and cnidocytes in *Nematostella* and *Hydractinia echinata* suggests that this *SoxB2*/*PaxA* pathway may well be conserved across cnidarians.

## Background

Cnidocytes (stinging cells) are a cnidarian-specific cell type and an important diagnostic feature of cnidarians (jellyfish, corals, hydroids, etc.). Used in prey capture, defense, and locomotion, cnidocytes are epithelial cells that contain an extrusive organelle (the cnidocyst) that varies widely in both form and function across cnidarians. Two major types of cnidocyte are recognized: *Nematocytes* contain a piercing/penetrating organelle, whereas *spirocytes* and *ptychocytes* contain adhesive/ensnaring organelles. While nematocytes are found in all cnidarians, spirocytes and ptychocytes are restricted to anthozoans (corals, sea anemones, etc.). This distribution of cnidocyte types across Cnidaria suggests that the ancestral cnidocyte was a nematocyte and that spirocytes and ptychocytes may have evolved by modification from this ancestral nematocyte in the lineage leading to Anthozoa [1]. While cnidocyte development has been fairly well characterized in hydrozoan cnidarians, other cnidarian lineages have received relatively little attention, making it difficult to find commonalities across cnidarians.

Studies of *Hydra* (Medusozoa:Hydrozoa) have revealed much about the cell dynamics of cnidogenesis in this organism [2, 3]. In *Hydra*, nests of new nematocytes differentiate synchronously from a population of hydrozoan-specific progenitor cells called interstitial cells that are found throughout the mid-gastric region of the ectoderm. The cnidocyst develops in a post-Golgi vacuole during terminal differentiation of the cnidocyte from its interstitial cell progenitor [reviewed by 4]. The proteins that comprise the structural elements of the cnidocyst (tubule, harpoon, and capsule wall) are largely cnidocyte specific and include the minicollagens and nematogalectins [5–8]. These proteins undergo posttranslational modifications including cleavage from a preproprotein, alternative splicing, and extensive disulfide bonding to form the contiguous structure of the tubule/capsule [6, 9, 10]. Initial synthesis of the structures comprising the cnidocyst is followed by invagination of the eversible tubule and swelling of the capsule to generate the high intracapsular osmotic pressure that enables rapid discharge of the mature organelle [11]. This final step of cnidocyst maturation requires the synthesis of poly-γ-glutamate by enzymes found within the developing capsule [12–14]. The enzyme thought to be responsible for the synthesis of poly-γ-glutamate, γ-glutamyl transpeptidase (*Ggt*), has been isolated from discharged cnidocytes from hydrozoan, anthozoan, and cubozoan lineages, suggesting that *Ggt* may also be a ubiquitous marker of cnidocytes [7, 13, 15, 16]. Further surveys of gene and protein expression in cnidocytes from across cnidarians have revealed that these novel cells are highly heterogeneous, expressing both novel (cnidarian-specific) and conserved genes [7, 9, 15, 17–20].

**Fig. 1:**
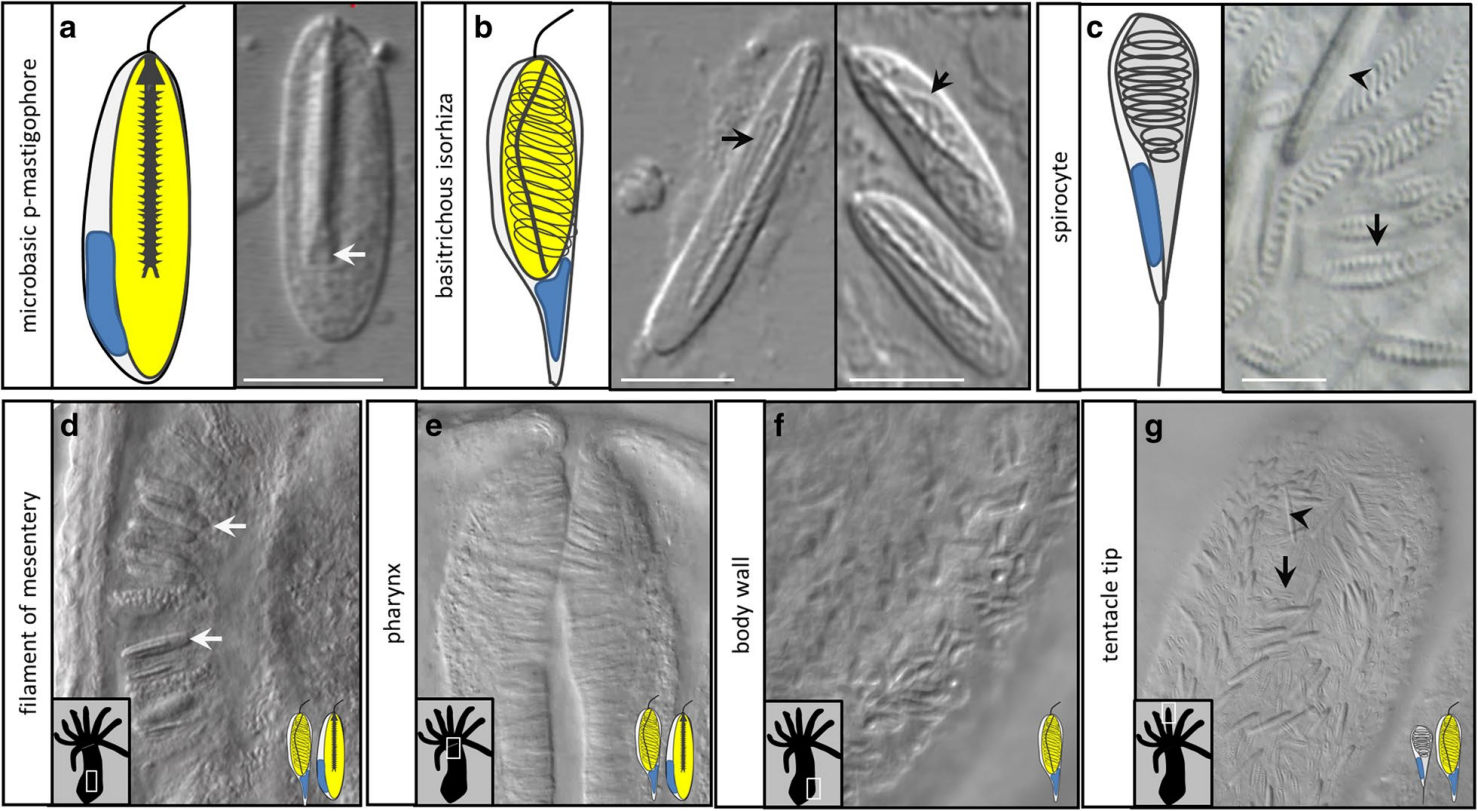
Type and distribution of cnidocytes in *N. vectensis*. **a** Microbasic p-mastigophores have a thick shaft which ends in a distinctive v-shaped notch (*arrow*). **b** Basitrichous isorhizas are variable in size (compare *left* and *right* DIC images) and have a thin shaft lacking a v-shaped notch and a tubule which is visible through the capsule (*arrows*). **c** Spirocytes can be identified by the regularly spaced coiled tubule and the lack of a visible capsule (*arrow*); a basitrichous isorhiza is shown for comparison (*arrowhead*). **d**, **e** The mesenterial filaments and pharynx are populated largely by microbasic p-mastigophores and large basitrichous isorhizas. **f** The body wall is populated by small basitrichs, and **g** the tentacle tips are populated by large basitrichous isorhizas (*arrowheads* in **c**, **g**) and spirocytes (*arrows* in **c**, **g**). Only nematocytes (basitrichous isorhizas and microbasic p-mastigophores) can be labeled using DAPI, indicated by *yellow color* in the cartoons (**a**, **b**). *Scale bars* in **a**–**c** represent 5 μm. All images are DIC micrographs

Cnidocyte development has received relatively little study in anthozoans, as compared with hydrozoans, and these studies have focused largely on the sea anemone, *Nematostella vectensis* [21, 22]. *N. vectensis* is reported to have three types of cnidocyte (Fig. 1a–c): two types of nematocyte (basitrichous isorhizas and microbasic p-mastigophores) and spirocytes. Two different size classes of basitrichous isorhizas have been identified [23, 24], though it is unclear whether size variation correlates with functional variation in this cell type. The distribution of cnidocytes in *N. vectensis* varies by tissue [23–26], and adult tissues in *N. vectensis* are populated by multiple different types of cnidocyte at the same time (Fig. 1d–g). Examination of the spatiotemporal distribution of minicollagens in *N. vectensis* revealed that cnidocyte development begins early, coincident with the onset of gastrulation [22]. In contrast with hydrozoan models, cnidocytes in *N. vectensis* appear to differentiate locally, in the tissue where they are deployed, from a dispersed population of proliferating progenitor cells [27].

Transcription factors in the *Sox* (SRY-related HMG box) family are known to play diverse roles in the development and maintenance of cell identity [28]. A recent study of neurogenesis in *N. vectensis* revealed that transcription factor *SoxB2*, an ortholog of bilaterian SoxB genes [29], is expressed in a population of progenitor cells that give rise to both neurons and cnidocytes and that knockdown of *SoxB2* results in loss of minicollagen-expressing cells [30]. The transcription factor *Mef2* (myocyte enhancer factor 2) is also suspected to play a role in cnidogenesis in *N. vectensis* as knockdown of *Mef2* splice variant IV (*Mef2IV*) also resulted in loss of minicollagen-expressing cells [31], but the relationship between *SoxB2* and *Mef2IV* has not been characterized. Although several other genes have been suggested to play a role in cnidocyte development based on the timing and distribution of their mRNA expression, there have been no further studies of the gene regulatory network controlling cnidocyte development in *N. vectensis*.

Paired box (*Pax*) genes are a family of homeodomain transcription factors that play an important role in specifying sensory cell identity of diverse metazoan tissues [32]. Perhaps, the most well-studied among all *Pax* genes, *Pax6*, is known to play an essential role in both the development and maintenance of the photosensitive tissues including the eye in bilaterians [33]. The presence of *Pax* orthologs in ctenophores [34, 35], sponges [36–39], and placozoans [40, 41] and their absence from the choanoflagellate *Monosiga brevicollis* [42], suggests that this gene family evolved in the stem metazoan, long before the origin of eyes, though paired-like genes may have a more ancient origin [43]. Cnidarians possess orthologs of all *Pax* lineages found in bilaterians except *Pax4*/*6* [42], suggesting that diversification of this gene family largely occurred before the common ancestor of cnidarians and bilaterians. Additionally, cnidarians appear to have undergone several lineage-specific duplication events, resulting in the cnidarian-specific *PaxA* and *PaxC*, and the multiple orthologs of *PaxD* found specifically in *N. vectensis* [44]. The function of *Pax* genes has been studied in several cnidarian taxa, both with and without eyes [41, 42, 45, 46]. *PaxB*, an ortholog of *Pax2*/*5*/*8*, has been shown to play a vital role in the development of the camera-type eye in the cubozoan *Tripedalia cystophora* [45], and *PaxA* has been shown to regulate the development of the simple eyes of the hydrozoan *Cladonema radiatum* [42]. The genome of the scyphozoan *Aurelia aurita* encodes two *Pax* genes (*PaxA* and *PaxB*), neither of which seems to be involved in the development of the eyes in this species [47].

Seven *Pax* genes have been described from *N. vectensis*: *PaxA*, *PaxB*, *PaxC*, *PaxD1*–*D4* [44, 48]; based on their expression pattern, *PaxA* and *PaxC* have been hypothesized to play a role in the development of neurons and/or cnidocytes [44]. Using a combination of descriptive and functional techniques, we test the hypothesized role of *PaxA* and *PaxC* in the development of cnidocytes in *N. vectensis*. We show that *PaxA* is expressed exclusively in developing cnidocytes, that knockdown of *PaxA* results in decreased expression of many cnidocyte-specific genes, and that co-expression of *Mef2IV* in a subset of the *PaxA*-expressing cells defines a second lineage of developing cnidocyte. Together, these results suggest spatiotemporal variability in the expression of multiple conserved transcription factors may be responsible for generating cnidocyte morphological and functional diversity.

## Results

### Cnidogenesis occurs continuously throughout the ectoderm in *N. vectensis*

**Fig. 2:**
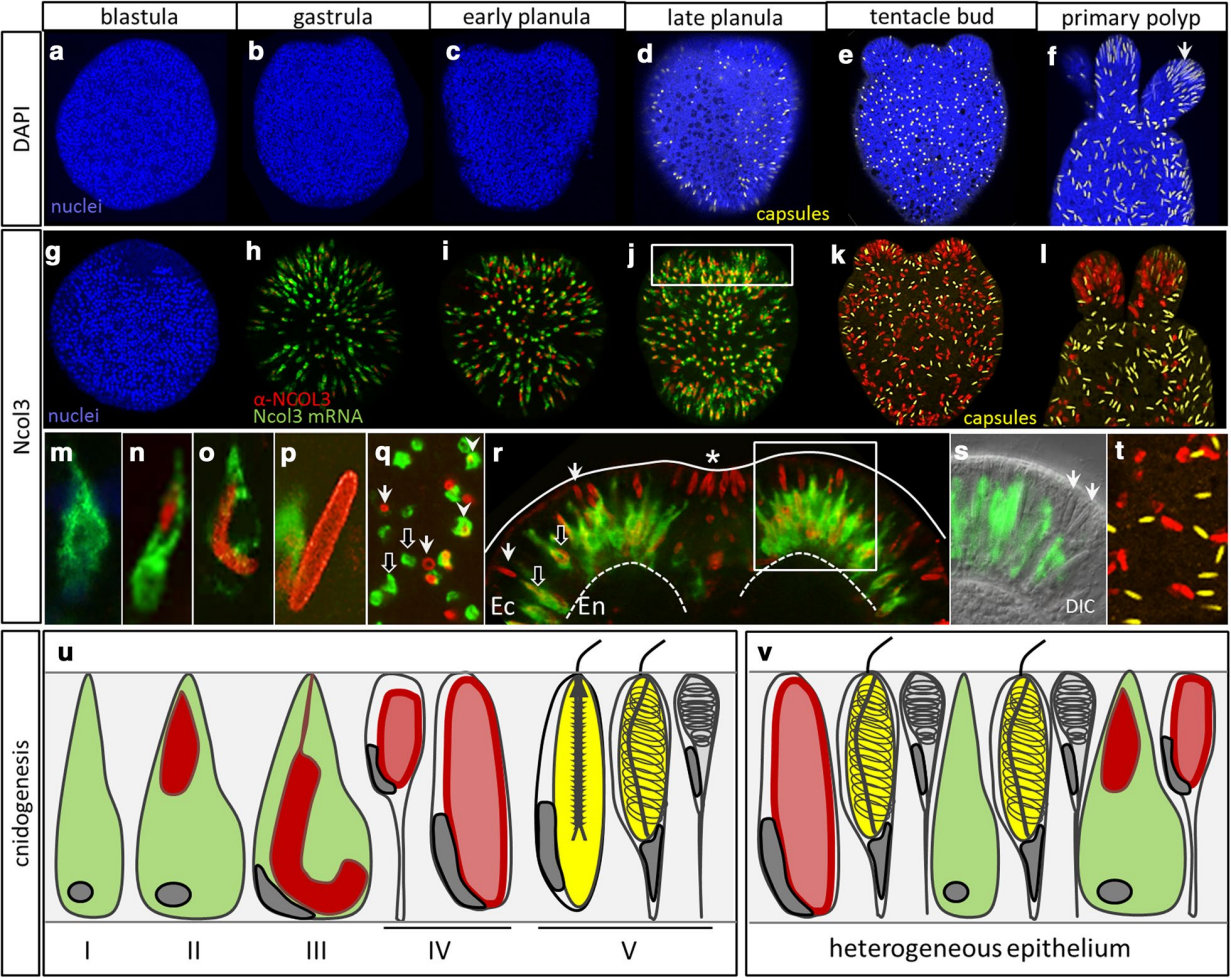
Stages of cnidogenesis in *N. vectensis*. **a**–**f** Mature nematocyst capsules (*yellow*, DAPI) are first detected in the ectoderm in the late planula stage and are dense in the tentacle tips at the polyp stage (**f**, *arrow*). **g**–**l** Expression of *Ncol3* mRNA (*green*) and NCOL3 protein (*red*) at the gastrula stage (**h**). New cnidocytes continue to develop in the embryonic ectoderm at all stages, even when mature cnidocytes (*yellow*, DAPI) are present in this tissue (**k**, **l**). **m**–**s** Multiple stages of cnidocytes are present in the ectoderm at the same time. Early-stage cnidocytes express *Ncol3* mRNA only (**m**); later, many developing cnidocytes express mRNA and translated protein at the same time (**n**, **o**). Late-stage cnidocytes express protein only (P) and appear like hollow cylinders (*arrows*, **q**). **r** In the late planula, developing cnidocytes are dense in the ectoderm (Ec) adjacent to the blastopore (*asterisk*). Early-stage cnidocytes (*black arrows*) appear shifted toward the mesoglea side of the ectoderm (*dotted line*), and late-stage cnidocytes (*white arrows*) appear shifted toward the apical side of the ectoderm (*solid line*). (**r** corresponds to the boxed region in **j**.) **s** Cells expressing *Ncol3* mRNA only continue to develop in the embryonic ectoderm, even in the presence of mature cnidocytes (*arrows*, **s**). (**s** corresponds to the boxed region in **r**). **t** NCOL3 protein (*red*) and DAPI (*yellow*) do not co-localize at any stage of development. **u** Cartoon summary of cnidocyte development as observed through *Ncol3* and DAPI labeling. *Colors* correspond to the labeling in **a**–**t**. Nuclei are *blue*, DAPI. The blastopore/oral pole is oriented to the top unless otherwise indicated by *asterisk* for this and all subsequent figures. Images in **a**–**l** are 3D renderings from confocal z-stacks; images **m**–**t** are single optical sections

**Fig. 3:**
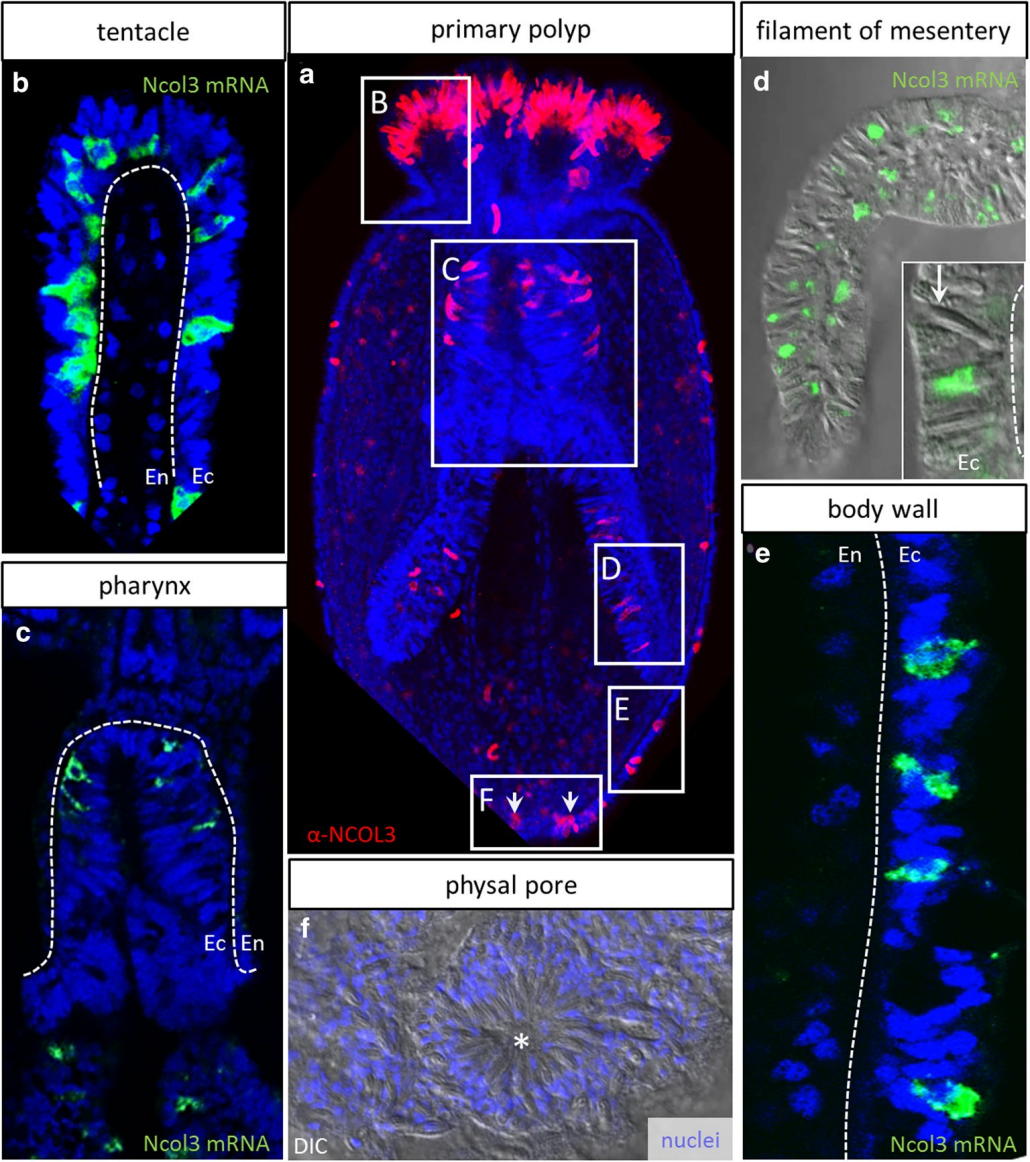
Distribution and development of new cnidocytes in tissues of the polyp. **a** Four-tentacle primary polyp illustrating the development of new cnidocytes (NCOL3 protein, *red*) in the ectoderm of multiple tissues. **b**–**e** Individual tissues from eight-tentacle-stage polyps showing expression of *Ncol3* mRNA (*green*) in the ectodermally derived tissue (Ec) only (*dotted line* indicates mesoglea; En—endodermally derived tissue). The *arrow* in **d** points to a mature cnidocyte in the cnidoglandular (ectodermal) portion of the mesenterial filament. **f** Aggregation of mature cnidocytes encircling an ephemeral opening (*asterisk*) at the aboral pole of the adult polyp. The *arrows* in **a** correspond to new cnidocytes developing in the region highlighted in **f**. **a** 3D rendering from a confocal z-stack; **b**, **c**, **e** individual optical sections; **d**, **f** DIC micrographs

To study the differentiation of cnidocytes in situ, we used a combination of markers that label distinct stages of cnidocyst development (Fig. 2). RNA probes were used to label cells undergoing transcription of minicollagen (*Ncol*), and antibodies were used to label cells that have progressed past the onset of NCOL protein translation, but have not yet completed polymerization of the developing capsule, at which time mature cnidocysts are no longer recognized by NCOL antibodies [22]. High-concentration (143 μM) DAPI was used to label poly-γ-glutamate in the matrix of the mature cnidocyst capsule [14]. Cnidocytes develop early during embryogenesis in *N. vectensis*, and new cnidocytes continue to develop exclusively in the ectoderm of all life stages (Fig. 2, 3). We confirm earlier reports that mature cnidocyst capsules first appear in the late planula stage of development and are abundant throughout the ectoderm at all stages thereafter (Fig. 2a–f) [21]. Cnidocyte differentiation, measured by the onset of expression of *Ncol3* mRNA, however, begins much earlier [22]. Co-localization of *Ncol3* mRNA and NCOL3 protein indicates that initial transcription of *Ncol3* starts before the gastrula stage and continues throughout development in the ectoderm only (Fig. 2g–t). In gastrula-stage embryos, early-stage cnidocytes expressing *Ncol3* mRNA only and cells that have already begun translation of NCOL3 protein are both present throughout the ectoderm (Fig. 2g, h, m–o), suggesting that the onset of *Ncol3* transcription occurs before gastrulation. In the early and late planula stages, many cells have downregulated expression of mRNA and are labeled with only α-NCOL3 antibody (Fig. 2i, j, p–r). Co-labeling with α-NCOL3 antibody and DAPI in the later stages (Fig. 2k, l, t) confirms that these two markers label distinct stages of cnidogenesis as no cnidocytes were ever labeled with both probes at the same time. Higher-magnification images illustrate the stages of capsule morphogenesis (Fig. 2m–p) and clearly show that developing cnidocytes progress through a curved U- or J-shaped stage as previously demonstrated for another sea anemone, *Metridum senile* [49]. Viewed from the surface of the ectoderm (perpendicular to the long axis of the developing cnidocyst), it is clear that cnidocytes develop asynchronously throughout the heterogeneous epithelium, such that adjacent cells are often at very different stages of cnidogenesis (Fig. 2q). Furthermore, although this is a simple epithelium composed of a single cell layer, early-stage cnidocytes appear shifted toward the basal side of the epithelium as RNA expression localizes basally (Fig. 2r, s). By contrast, late-stage cnidocytes lacking mRNA expression appear shifted toward the apical membrane as capsule maturation occurs apically. The different morphological stages of cnidogenesis and a stylized representation of the heterogeneous embryonic epithelium are presented in Fig. 2u, v. Using both α-NCOL3 antibody (Fig. 3a) and *Ncol3* mRNA probe (Fig. 3b–e), we demonstrate that cnidogenesis continues into the polyp stage exclusively in the ectodermal component of polyp tissues: tentacles (Fig. 3b), pharynx (Fig. 3c), mesenterial filaments (Fig. 3d), and body wall (Fig. 3e). We also demonstrate a ring of mature cnidocytes encircling the physal pore (Fig. 3f), an ephemeral opening in the aboral end of the polyp, which has not yet been described.

### Minicollagens are spatially co-expressed but may vary temporally

**Fig. 4:**
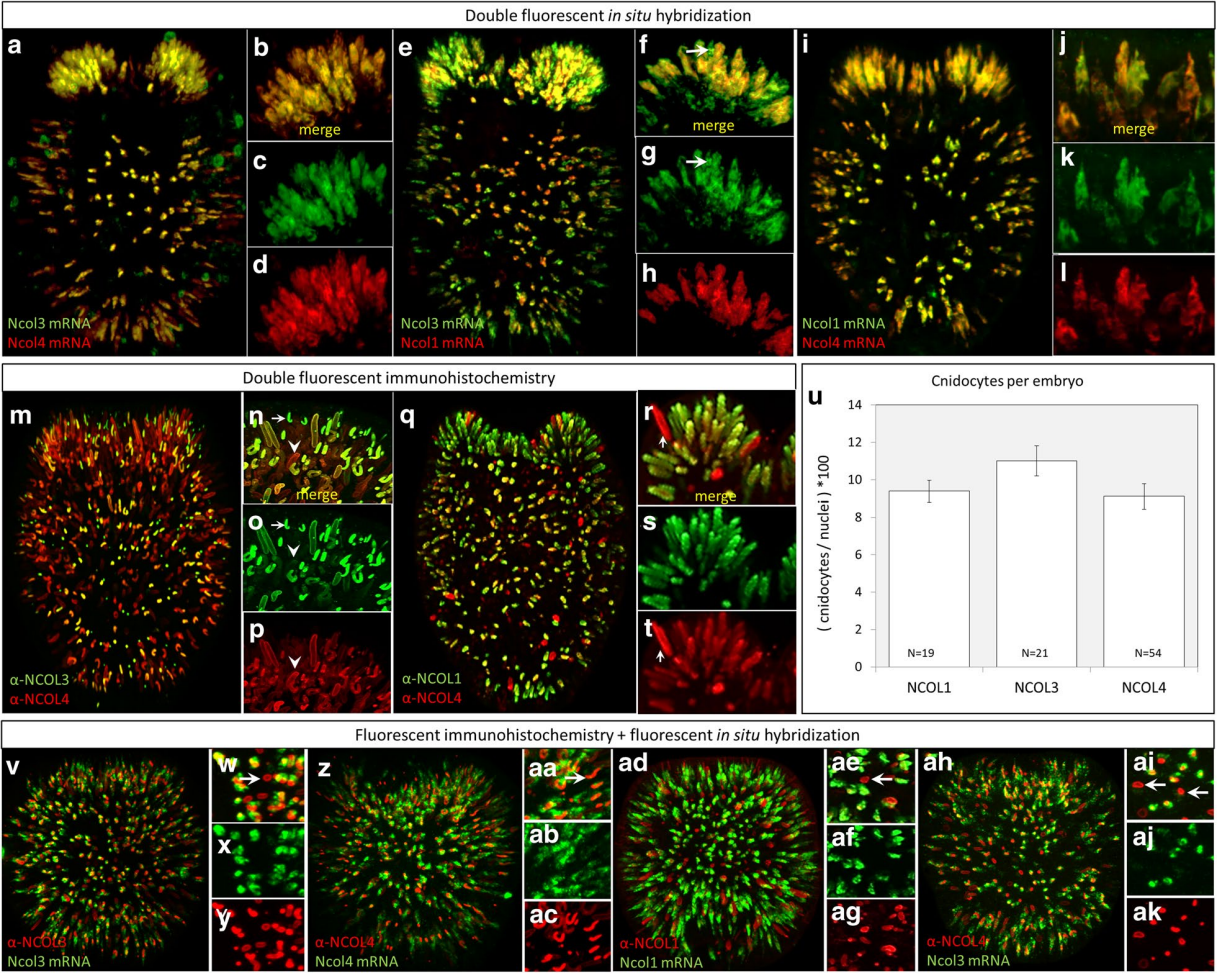
Minicollagen mRNAs are co-expressed, but the onset of NCOL protein localization is variable. **a**–**d** *Ncol3* and *Ncol4* mRNAs are co-expressed in all developing cnidocytes at the tentacle bud stage. **e**–**h** *Ncol3* and *Ncol1* mRNAs are also largely co-expressed, though cnidocytes that appear to be expressing *Ncol3* exclusively can be seen in the ectoderm (*arrows* **f**, **g**). **i**–**l** *Ncol4* and *Ncol1* mRNAs are co-expressed in all cnidocytes. **m**–**p** NCOL3 and NCOL4 proteins largely co-localize, though early-stage capsules may express only NCOL3 (*arrows* **n**, **o**) and late-stage capsules may express only NCOL4 (*arrowheads* **o**, **p**). **q**–**t** NCOL1 and NCOL4 proteins also co-localize in all developing cnidocytes, though some late-stage capsules may express only NCOL4 (*arrows* **r**, **t**). **u** In gastrula-stage embryos, cnidocytes comprise approximately 10% of the cells, independent of which antibody is used (NCOL1: 9.39 ± 0.59, NCOL3: 11.01 ± 0.81, NCOL4: 9.11 ± 0.68; mean ± standard error). *N* = number of embryos analyzed. (V-AG) Co-labeling with mRNA probes and antibodies for the corresponding protein confirms that transcription is downregulated before cnidocyst capsules are fully polymerized as proteins can be detected in cells that no longer express mRNA for each antibody/probe combination (*arrows* **w**, **aa**, **ae**). **ah**–**ak** Co-labeling of *Ncol3* mRNA with α-NCOL4 also reveals many late-stage cnidocytes labeled with α-NCOL4 only (*arrows* **aj**). Main images are 3D renderings from confocal z-stacks, high-magnification panels are single optical sections

The co-expression of minicollagens was examined by double-fluorescent in situ hybridization (dFISH; Fig. 4a–l), double-fluorescent immunohistochemistry (dFIHC; Fig. 4m–t), and a combination of the two techniques (FISH/FIHC, Fig. 4v–ak). *Ncol3* and *Ncol4* mRNAs are co-expressed in all developing cnidocytes of the body wall and budding tentacles (Fig. 4a–d); we therefore use Ncol3 and Ncol4 as ubiquitous markers of cnidocyte development throughout the rest of this study. *Ncol1* mRNA appears to be expressed in only a portion of the cnidocytes expressing *Ncol3* mRNA in the body wall and tentacle ectoderm (Fig. 4e–h), whereas *Ncol1* and *Ncol4* appear to be fully co-expressed in both tissues (Fig. 4i–l). NCOL3 and NCOL4 proteins largely co-localize (Fig. 4m–p), although several early-stage cnidocytes lack labeling for NCOL4 (Fig. 4n), while several late-stage cnidocytes lack labeling for NCOL3 (Fig. 4p). Similarly, NCOL4 and NCOL1 largely co-localize (Fig. 4q–t) although several NCOL4-labeled late-stage cnidocytes seem to lack NCOL1 labeling (Fig. 4r). Using Imaris software (Bitplane, Concord, MA, USA), we counted the number of cells that were labeled with α-NCOL1, α-NCOL3, and α-NCOL4 antibodies at the planula stage and found approximately equal numbers of cells were labeled with each antibody (Fig. 4u). Co-labeling with probe and antibody to detect mRNA and protein at the same time (Fig. 4v–ak) confirms the morphological staging of cnidocyte development demonstrated in Fig. 2 for all three minicollagens.

### Nematogalectin, not γ-glutamyl transpeptidase, is a ubiquitous marker of cnidogenesis

**Fig. 5:**
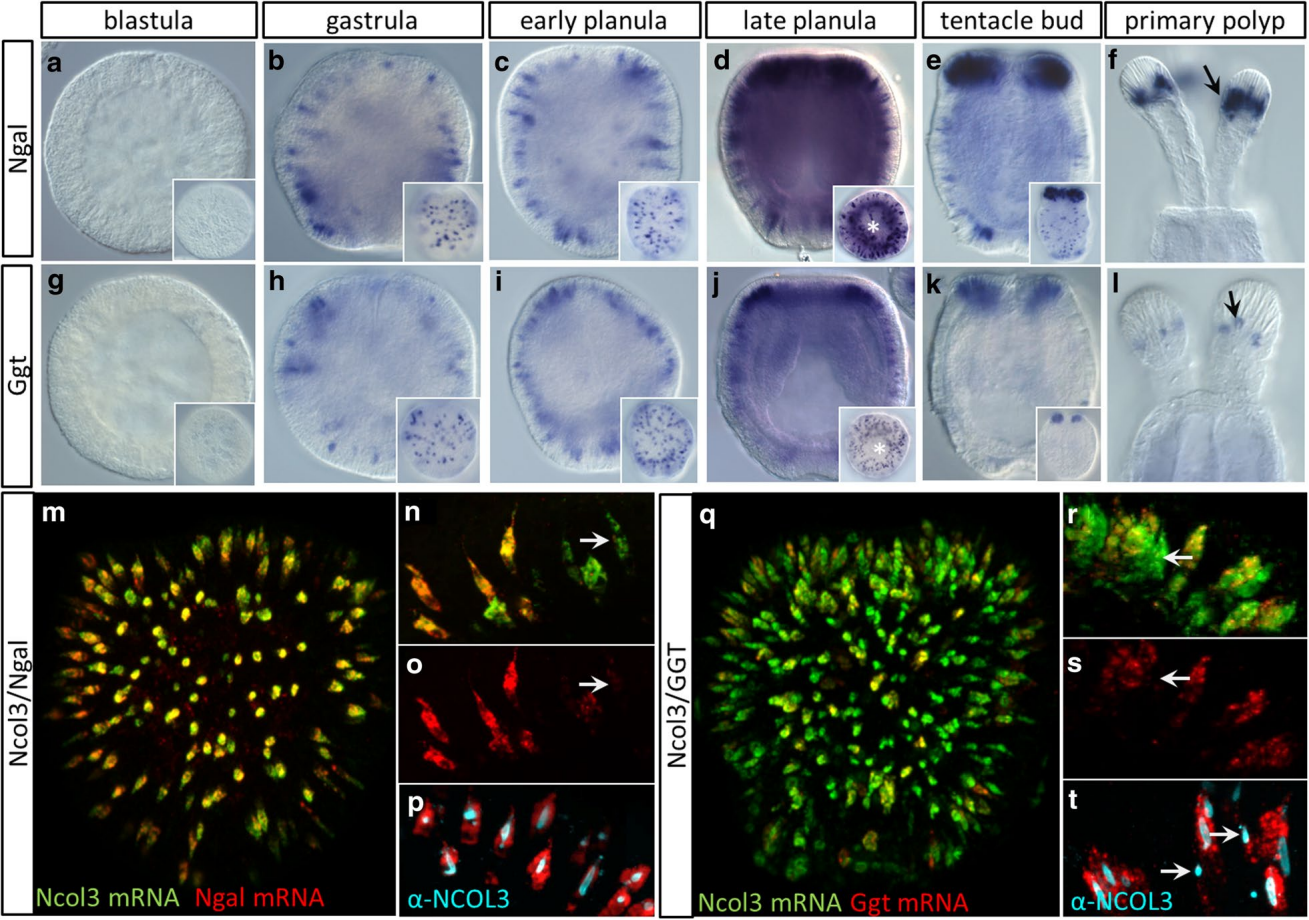
Characterization of two novel markers for differentiated cnidocytes. Expression of **a**–**f** nematogalectin (*Ngal*) mRNA and **g**–**l** γ-glutamyl transpeptidase (*Ggt*) mRNA during development. Onset of expression begins at/before the gastrula stage in scattered cells of the ectoderm (surface plane, *insets*) for both *Ngal* (**b**) and *Ggt* (**h**). *Ngal* is expressed in the body wall ectoderm throughout development (**b**–**e**, *insets*), and expression becomes dense in the developing tentacle buds (**e**) and tentacle tips of the polyp (**f**, *arrow*). *Ggt* is also expressed in the embryonic ectoderm (**h**–**j**) and presumptive tentacle buds but is absent from the body wall at later stages (**k**, *inset*) and is expressed in only few cells of the tentacle tips (**l**, *arrow*). **m** *Ngal* mRNA is co-expressed with *Ncol3* mRNA in most developing cnidocytes in early-planula-stage embryos. **n**, **o** Few cells exhibit *Ncol3* mRNA expression without *Ngal* (*arrows*), but **p** *Ngal* mRNA and NCOL3 protein are perfectly co-expressed. **q**–**s** *Ggt* mRNA is co-expressed with *Ncol3* mRNA in many but not all developing cnidocytes. (*Arrows* in **r**–**s** indicate cells expressing *Ncol3* only.) **t** Co-labeling of NCOL3 protein and *Ggt* mRNA confirms that some cnidocytes express *Ncol3* without *Ggt* (*arrows*). Images in **a**–**l** are DIC micrographs, images **m** and **q** are 3D renderings from confocal z-stacks, and images **n**–**p** and **r**–**t** are individual optical sections

To identify other markers of terminally differentiated cnidocytes and compare the molecular nature of cnidocytes from anthozoans and hydrozoans, we searched the list of 51 nematocyte-specific genes identified from *Hydra* [20] for orthologs in *N. vectensis*. From this search, we identified two genes with expression patterns suggestive of a role in cnidogenesis: nematogalectin (*Ngal*; Nemve1|232015) and γ-glutamyl transpeptidase (*Ggt;* Nemve1|100407) (Fig. 5). Both genes were expressed in scattered ectodermal cells of embryos of all stages following gastrulation (Fig. 5a–l). At later stages the expression patterns of these two genes differ: In addition to the abundant expression of *Ngal* in the tentacle buds and mature tentacles (Fig. 5e, f), this gene is also expressed in the body wall ectoderm (inset, Fig. 5e), whereas *Ggt* expression is restricted to the tentacles in both stages (Fig. 5k, l). *Ngal* mRNA is co-expressed with *Ncol3* mRNA in many, but not all cells (M–O); however, all cells expressing *Ngal* mRNA are also labeled with α-NCOL3 antibody (Fig. 5p). These results suggest that *Ncol3* and *Ngal* are both ubiquitous markers of cnidocyte development, but the former is transcribed earlier than the latter. *Ggt* and *Ncol3* are also co-expressed in many, but not all, developing cnidocytes (Fig. 5q–s), but co-labeling with *Ggt* mRNA and α-NCOL3 reveals a population of cells that express NCOL3 protein without *Ggt* mRNA. Thus, *Ggt* appears to be expressed in only a subset of cnidocytes.

### Knockdown of SoxB2 results in decreased expression of all cnidocyte markers

**Fig. 6:**
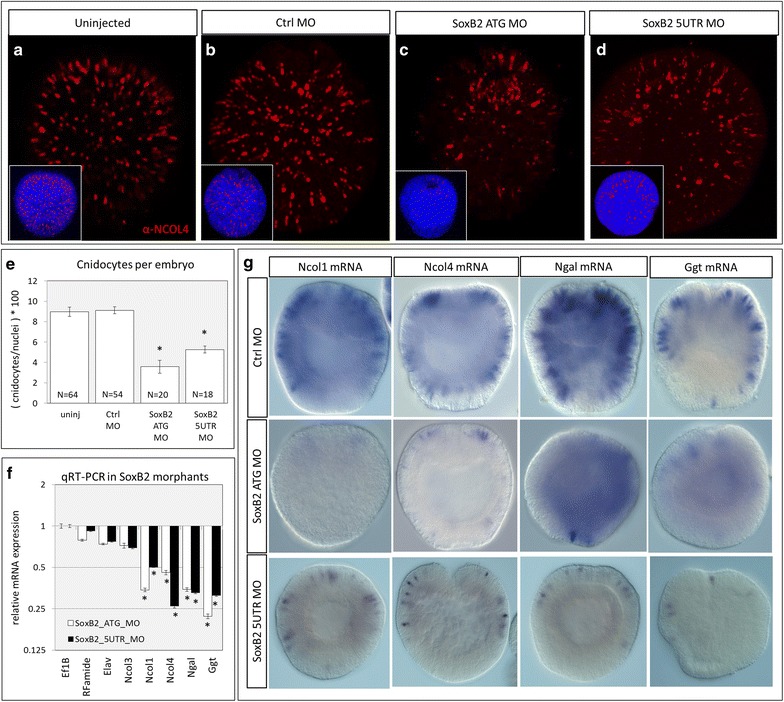
Identification of putative cnidocyte-specific transcription factors. **a**–**d** Cnidocyte abundance was characterized in gastrula-stage embryos labeled with α-NCOL4 antibody in **a** unmanipulated embryos or following microinjection of **b** standard control morpholino (Ctrl MO), **c** SoxB2 ATG MO, or **d** SoxB2 5UTR MO. Nuclei were labeled with DAPI (*insets*), and cnidocyte abundance was measured relative to the total number of labeled nuclei in each embryo. **e** Cnidocytes comprise approximately 9% of the cells of the ectoderm in uninjected and control MO-injected embryos and only 4% of the cells in SoxB2 MO embryos (uninjected: 8.98 ± 0.45, control MO: 9.11 ± 0.33, SoxB2 ATG MO: 3.82 ± 1.84, SoxB2 5UTR MO: 5.27 ± 0.34; mean ± standard error). *Asterisk* indicates significant difference from control MO-injected embryos. **f** Relative mRNA expression of target genes assayed by qRT-PCR following microinjection of *SoxB2* MO. The expression of housekeeping gene EF1B in *SoxB2* morphants relative to embryos injected with control MO was set to 1.0, and all other expression values are reported relative to this. The *y*-axis is presented in log scale. Mean ± standard error. *Asterisk* indicates significant difference from Ef1B. **g** In situ hybridization of cnidocyte-specific genes in control MO-injected and *SoxB2* MO-injected embryos validating the results of qRT-PCR analysis. Images in **g** are DIC micrographs

To characterize genes involved in the cnidogenesis pathway, we induced knockdown of *SoxB2* using two non-overlapping SoxB2 translation-blocking morpholinos (Table 1) and examined the effects on expression of cnidocyte-specific genes. Wild-type/uninjected embryos and embryos injected with a standard control morpholino (Ctrl MO; Table 1) express abundant NCOL4 protein in developing cnidocytes throughout the ectoderm at the gastrula stage (Fig. 6a–b). By contrast, those injected with translation-blocking morpholinos directed against the SoxB2 start site (SoxB2 ATG MO) or a region of the 5′ untranslated region (SoxB2 5UTR MO) had far fewer developing cnidocytes in the ectoderm (Fig. 6c, d). Quantification of these patterns revealed that cnidocytes comprise approximately 9% of the cells in uninjected and control morpholino-injected embryos (8.97 ± 3.8, 9.11 ± 1.3, respectively) and approximately 4–5% of the cells in *SoxB2* morphant embryos (ATG: 3.57 ± 1.84, 5UTR: 5.27 ± 0.33) (Fig. 6e). Using quantitative real-time PCR (qRT-PCR) in gastrula-stage embryos, we confirm that SoxB2 morphants exhibit a minor decrease in expression of two neural markers (*RFamide* and *Elav*) and one cnidocyte marker (*Ncol3*) as previously shown for the SoxB2 ATG MO [30]. Further, embryos injected with either SoxB2 ATG MO or SoxB2 5UTR MO exhibited significantly lower expression of all remaining cnidocyte markers examined (*Ncol1*, *Ncol4*, *Ngal*, and *Ggt)* compared to embryos injected with standard control morpholino (Fig. 6f). Using in situ hybridization, we validate the results of qRT-PCR analysis and demonstrate that only few cells expressing *Ncol1*, *Ncol4*, *Ngal*, or *Ggt* remain in the ectoderm following injection of either SoxB2 morpholino, relative to embryos injected with control morpholino (Fig. 6g). Together, these results confirm the role of *SoxB2* in regulating the differentiation of cnidocytes in *N. vectensis*, but suggest either some degree of inefficiency with *SoxB2*-mediated knockdown or, potentially, a *SoxB2*-independent cnidogenesis pathway, as we never observed a complete loss of cnidocytes in these experiments.

**Table 1:** Morpholinos used in this study

Morpholino	Type	Target^a^	Sequence (5′ → 3′)
Ctrl MO (0.9 mM)	Negative control	N/A	CCTCTTACCTCAGTTACAATTTATA
SoxB2 ATG MO (0.9 mM)	Translation-blocking	ATG	CATGCCCGTCTTCTTGCTTGCCCAT
SoxB2 5UTR MO (0.9 mM)	Translation-blocking	5′UTR	TATACTCTCCGCTGTGTCGCTA
PaxA sp MO (0.9 mM)	Splice-blocking	I1E2	AGGACCTTCAAGAACATTCGATAAT
PaxA tr MO (0.9 mM)	Translation-blocking	ATG	CCACCAGGACCTCTATGAGGCATAC
PaxC sp MO (0.6 mM)	Splice-blocking	E1I1	TCGCTCTGAATGCTTCTTACCTTCA
PaxC tr MO (0.6 mM)	Translation-blocking	ATG	GCCATAAGGAGTGGCCAGAAATCCT

^a^I1E2—targets the boundary between intron 1 and exon 2 (splice acceptor site) and E1I1—targets the boundary between exon 1 and intron 1 (splice donor site). ATG—targets the start codon. 5′UTR—targets the 5′ untranslated region. All morpholinos were designed by and purchased from GeneTools, LLC (USA)

### Knockdown of SoxB2 results in decreased expression of *Mef2IV* and *PaxA*, but not *PaxC*

**Fig. 7:**
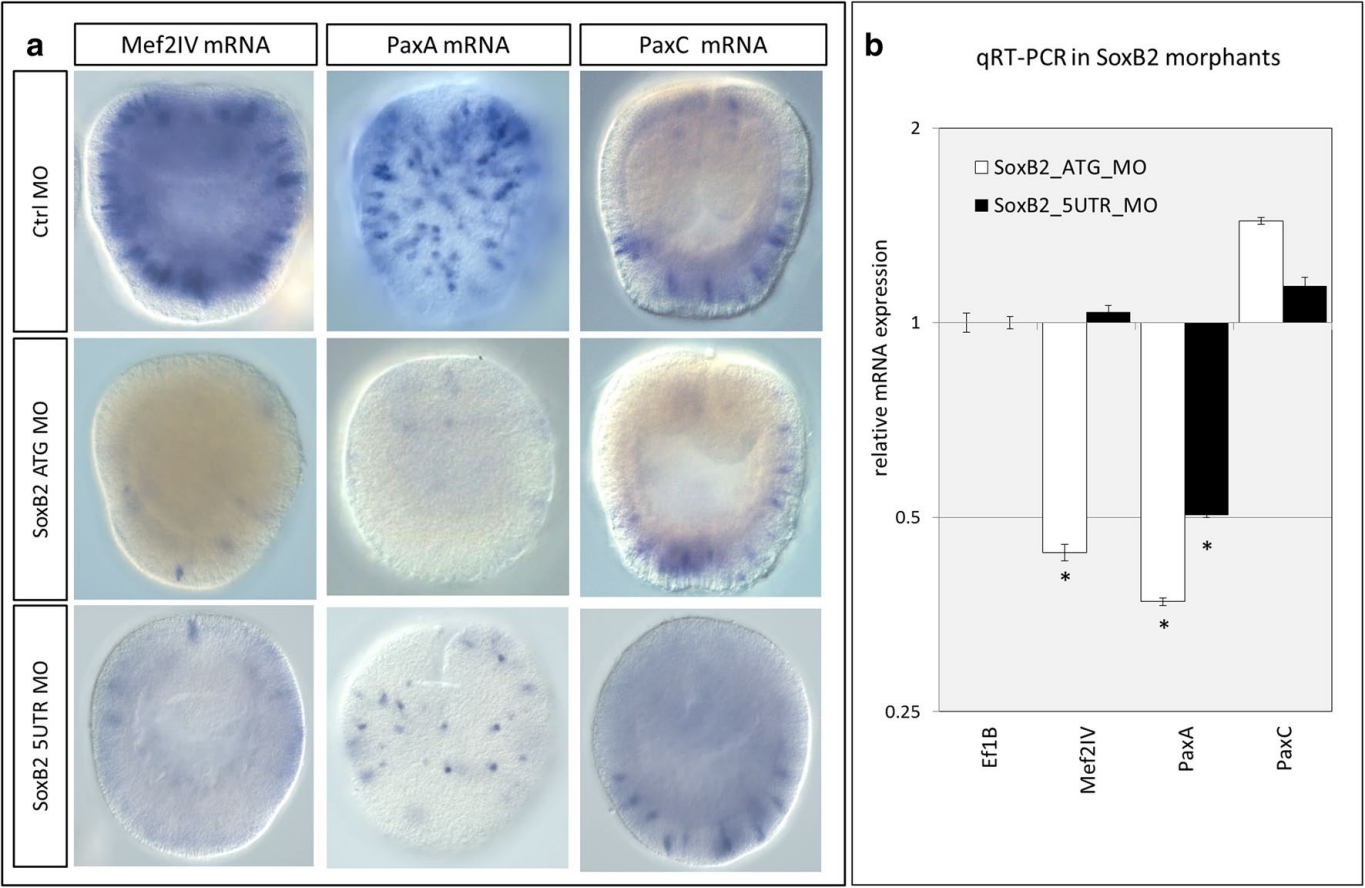
*Mef2IV* and *PaxA*, but not *PaxC*, are downstream of *SoxB2*. **a** Spatial expression of *Mef2IV*, *PaxA*, and *PaxC* in gastrula-stage embryos injected with control and SoxB2 morpholinos. **b** qRT-PCR in SoxB2 morphants demonstrates knockdown of *Mef2IV* (5UTR MO only) and *PaxA*, but not *PaxC*. *Asterisk* indicates significant difference from *Ef1B*. Images in **a** are DIC micrographs

Given that *SoxB2* regulates the differentiation of both neurons and cnidocytes in *N. vectensis* [30], we aimed to identify transcription factors that were specific to the cnidocyte differentiation pathway downstream of *SoxB2*. *Mef2IV* was previously shown to be expressed in scattered ectodermal cells of the gastrula-stage embryo [50], and a subsequent study of its function in vivo demonstrated that knockdown of *Mef2IV* by translation-blocking morpholino resulted in a decrease in the number of *Ncol3*-expressing cells [31]. Similarly, the expression of *PaxA* and *PaxC* during embryogenesis in *N. vectensis* is consistent with a role for these two transcription factors in patterning cnidocytes [44]; however, the function of these two genes has not been studied. Embryos injected with control morpholino exhibited wild-type expression of *Mef2IV*, *PaxA*, and *PaxC* in gastrula-stage embryos, whereas embryos injected with either SoxB2 morpholino exhibited fewer *Mef2IV*- and *PaxA*-expressing cells (Fig. 7a). The number of cells expressing *PaxC* did not appear to change in SoxB2 morphant embryos. Consistent with this, qRT-PCR results demonstrate a significant downregulation of *PaxA* in both treatments and a small, nonsignificant increase in *PaxC* expression in both treatments (Fig. 7b). Curiously, *Mef2* was downregulated in SoxB2 ATG morphants only, suggesting that the 5′ region of this transcript plays an important role in modulating transcription of the different splice variants. Thus, while *Mef2IV* and *PaxA* may be downstream of *SoxB2*, *PaxC* is likely expressed in a *SoxB2*-independent lineage of cells.

### *Mef2IV* and *PaxA*, but not *PaxC*, are co-expressed in differentiating cnidocytes

**Fig. 8:**
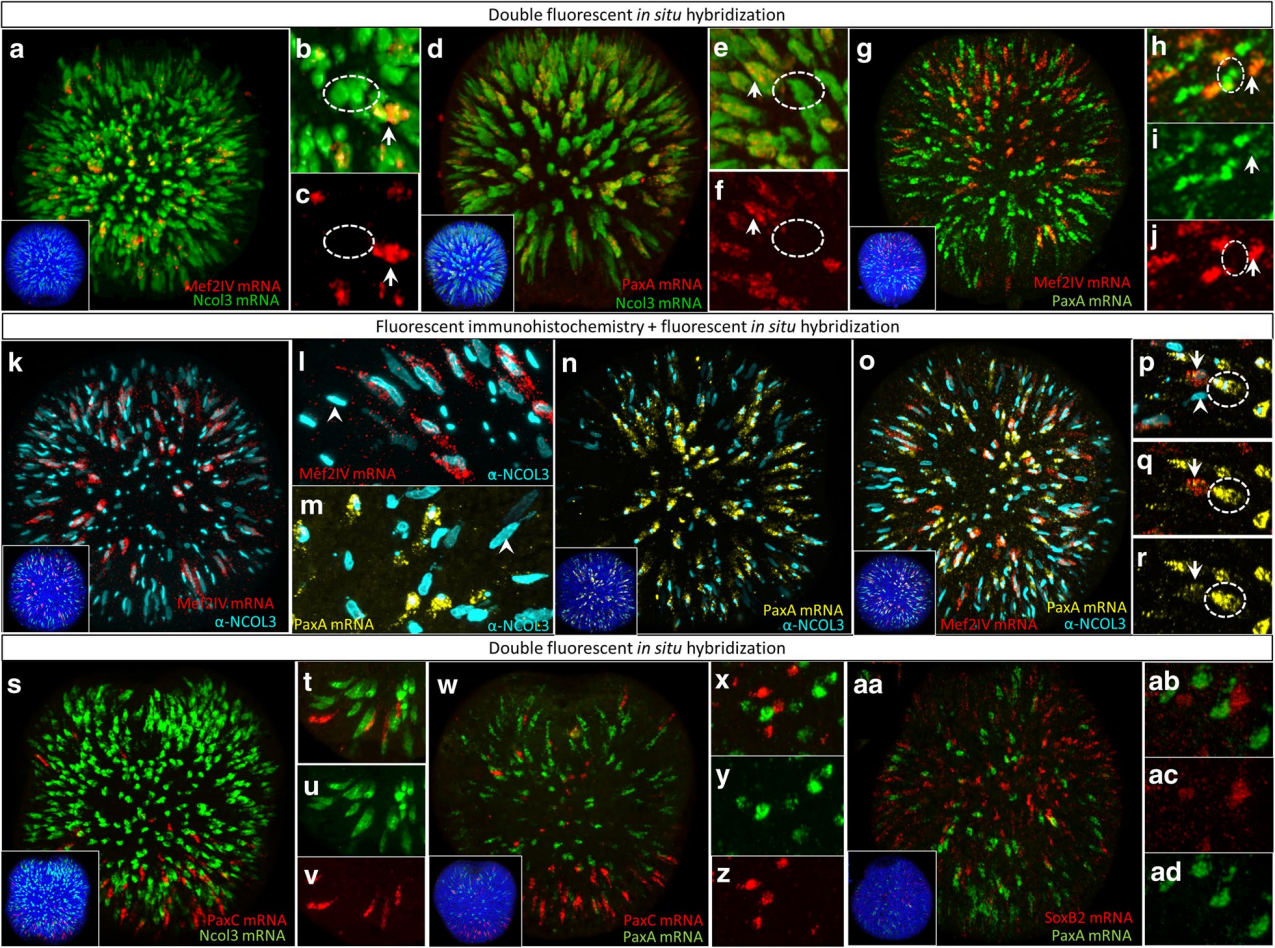
Co-expression of *Mef2IV* and *PaxA*, not *PaxC*, in developing cnidocytes. **a**–**c** *Mef2IV* mRNA co-localizes with *Ncol3* mRNA in only ~25% of the developing cnidocytes. (Cells co-expressing both markers are indicated by *arrows*, and cells expressing one marker only are indicated by *circles* in panels **a**–**j**). **d**–**f** *PaxA* mRNA co-localizes with *Ncol3* mRNA in ~50% of the developing cnidocytes. **g**–**j** *PaxA* mRNA is co-expressed in *Mef2IV*-expressing cells, but several cells express *PaxA* without *Mef2IV*. Co-localization of **k**, **l** *Mef2IV* mRNA and **m**, **n** *PaxA* mRNA with NCOL3 protein. **o**–**r** Co-labeling of *PaxA* and *Mef2IV* mRNA with α-NCOL3 indicates there are cells expressing all three markers at the same time (*arrows*), cells expressing *PaxA* and NCOL3 without *Mef2IV* (*dotted circles*), and cells expressing NCOL3 protein without either *PaxA* or *Mef2IV* (*arrowhead*). **s**–**v** *PaxC* is not co-expressed with *Ncol3* or **w**–**z** *PaxA*. **aa**–**ad** *PaxA* and *SoxB2* are not co-expressed. *Insets* and main images are 3D reconstructions from confocal z-stacks; high-magnification images are individual optical sections

We next examined the co-expression of *Ncol3* mRNA with *Mef2IV*, *PaxA*, and *PaxC* (Fig. 8). *Mef2IV* (Fig. 8a–c) and *PaxA* (Fig. 8d–f) are co-expressed with *Ncol3* in many developing cnidocytes, and co-labeling for both transcription factors shows that *Mef2IV*-expressing cells comprise a subset of the *PaxA*-expressing cells (Fig. 8g–j), whereby all *Mef2IV*-expressing cells also express *PaxA* and all *PaxA*-expressing cells also express *Ncol3*, but many *PaxA*-expressing cells lack *Mef2IV* expression and many *Ncol3*-expressing cells lack *PaxA* expression. Co-labeling of *Mef2IV* and *PaxA* with NCOL3 protein confirms the presence of cells expressing NCOL3 protein that do not express *Mef2IV* or *PaxA* (Fig. 8k–r). *PaxC* was never observed to be co-expressed with *Ncol3* (Fig. 8s–v) or *PaxA* (Fig. 8w–z). The morphology of the *PaxC*-expressing cells (elongated in the apico-basal axis; Fig. 8v) suggests these cells may be sensory cells. Finally, we examined the co-expression of *SoxB2* and *PaxA* during cnidogenesis and determined that *SoxB2* does not co-localize with *PaxA* at any time during development (Fig. 8aa–ad).

### Knockdown of *PaxA*, not *PaxC*, results in loss of cnidocytes

**Fig. 9:**
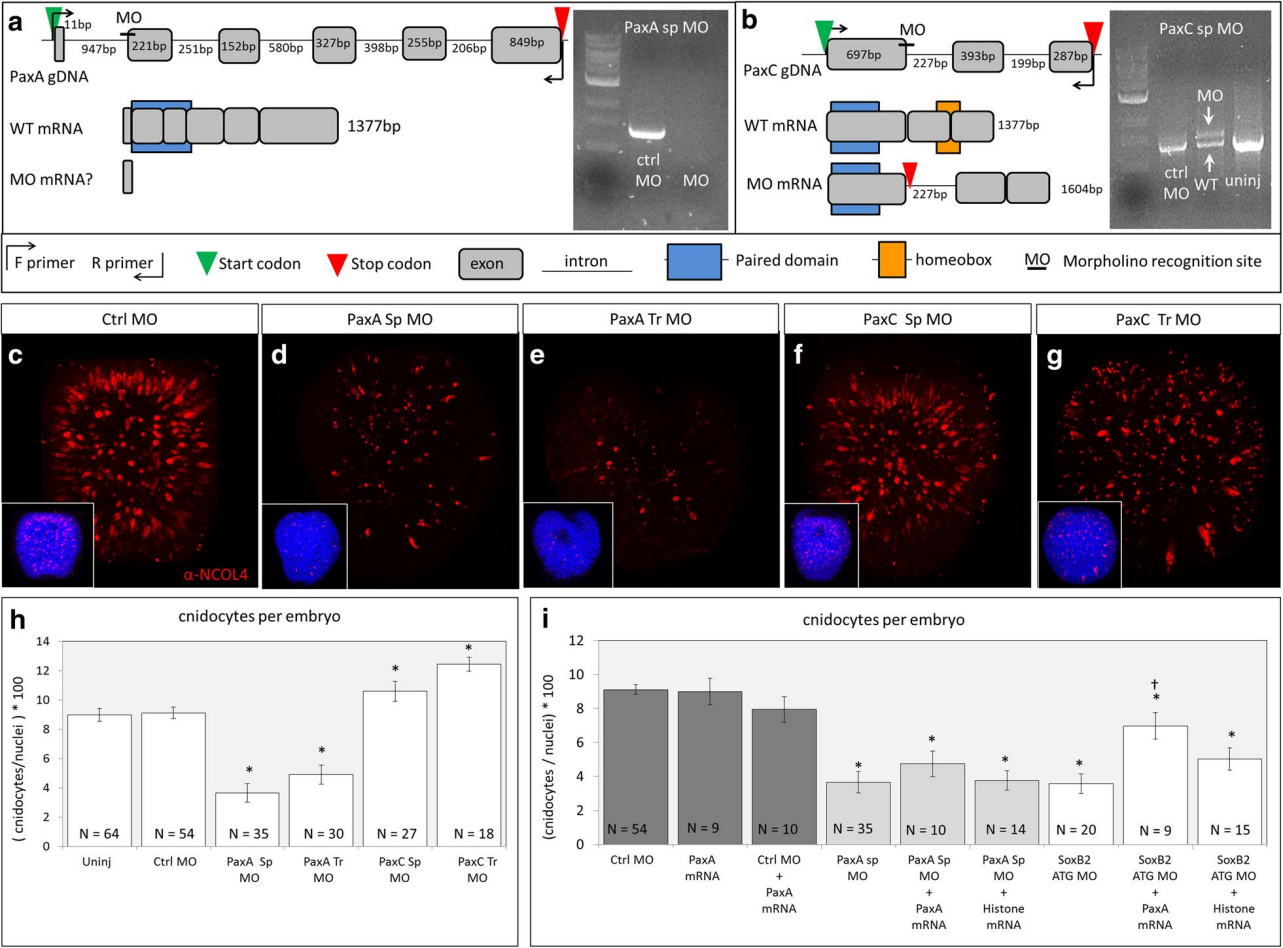
Knockdown of *PaxA* results in loss of cnidocytes. **a** The PaxA sp MO recognizes the I1E2 boundary which should cause skipping of exon 2, but the *PaxA* transcript could only be amplified from embryos injected with control morpholino (Ctrl MO). **b** The PaxC sp MO blocks the E1I1 boundary causing retention of intron 1. Both WT and morphant transcripts were amplified from PaxC MO-injected embryos. **c**–**g** Developing cnidocytes labeled with α-NCOL4 antibody in embryos injected with **c** control morpholino, **d** PaxA splice-blocking morpholino, **e** PaxA translation-blocking morpholino, **f** PaxC splice-blocking morpholino, and **g** PaxC translation-blocking morpholino. **h** Quantitative analysis of cnidocyte abundance in early-planula-stage embryos. Control morpholino-injected embryos exhibited wild-type abundance of cnidocytes, whereas PaxA sp morphants and PaxA tr morphants had significantly fewer (control MO: 9.11 ± 0.39, PaxA sp: 3.65 ± 0.63, PaxA tr: 4.90 ± 0.66; mean ± standard error). Both *PaxC* sp morphants and PaxC tr morphants exhibited an increase in cnidocyte abundance, albeit with a small effect size (PaxC sp: 10.58 ± 0.68, PaxC tr: 12.44 ± 0.47). **i** *PaxA* mRNA partially rescues SoxB2 ATG morphant phenotype but is not sufficient to induce ectopic cnidocytes. *Asterisk* indicates significant difference from Ctrl MO. *Dagger* indicates significant difference from *SoxB2* ATG MO

To assess the influence of *PaxA* and *PaxC* on cnidogenesis, we knocked down endogenous *Pax* expression using splice-blocking and translation-blocking morpholinos (Fig. 9). Morpholinos (Table 1) were microinjected into fertilized eggs before first cleavage, and embryos were reared to the early planula stage for analysis. The PaxA splice-blocking morpholino (PaxA sp MO) was designed to block the splice acceptor site at the boundary between intron 1 and exon 2 (I1E2 boundary). We first confirmed that transcribed mRNAs were improperly spliced by PCR amplification and sequencing of the region affected by the morpholino. Using PCR, we amplified the full 1.8 kb PaxA coding sequence from cDNA isolated from embryos injected with control morpholino (Fig. 9a); the same primer set failed to amplify a product from embryos injected with PaxA splice-blocking morpholino. The PaxC splice-blocking morpholino (PaxC sp MO) was designed to block the E1I1 boundary, and when injected, this morpholino results in retention of intron 1, which encodes a stop codon 12 amino acids downstream of the E1I1 boundary. The morphant *PaxC* transcript is therefore longer than the wild-type transcript and encodes a wild-type paired domain but the homeobox is no longer in the open reading frame (Fig. 9b). Gel electrophoresis confirms the presence of both transcripts in morphant embryos. To further support the efficacy of splice-blocking morpholinos, we performed tandem knockdown experiments using translation-blocking morpholinos for both PaxA and PaxC. Knockdown of *PaxA* (via splice- or translation-blocking morpholino) results in a decrease in the number of developing cnidocytes in the ectoderm of the planula-stage embryo (Fig. 9c–e), but knockdown of *PaxC* appears to have little effect on cnidocyte abundance (Fig. 9f, g). Quantification of these results indicates that the abundance of cnidocytes in the ectoderm of morphant embryos falls from approximately 9% in embryos injected with control morpholino to 4–5% in PaxA morphants (Fig. 9h). Surprisingly, quantitative analysis of *PaxC* knockdown reveals a small but significant increase in cnidocyte abundance such that the ectoderm of PaxC morphant embryos comprised 10–12% cnidocytes.

To test the sufficiency of *PaxA* to induce ectopic cnidocytes and rescue morpholino phenotypes we microinjected exogenous *PaxA* mRNA alone and in combination with morpholinos and assayed the effects on cnidocyte abundance in the ectoderm (Fig. 9i). Embryos injected with control morpholino only, *PaxA* mRNA only, or a combination of the two did not differ in cnidocyte abundance, suggesting *PaxA* is not sufficient to induce ectopic cnidocytes. The abundance of cnidocytes also did not increase in embryos injected with a combination of *PaxA* mRNA and PaxA sp MO as compared with those injected with PaxA sp MO alone, and neither of these treatments differed from embryos injected with PaxA sp MO and control mRNA encoding histone H2B. Conversely, embryos injected with *PaxA* mRNA in combination with SoxB2 ATG translation-blocking morpholino experienced a partial rescue as cnidocyte abundance increased significantly in this treatment relative to embryos injected with SoxB2 ATG MO alone and those injected with SoxB2 ATG MO and control mRNA.

**Fig. 10:**
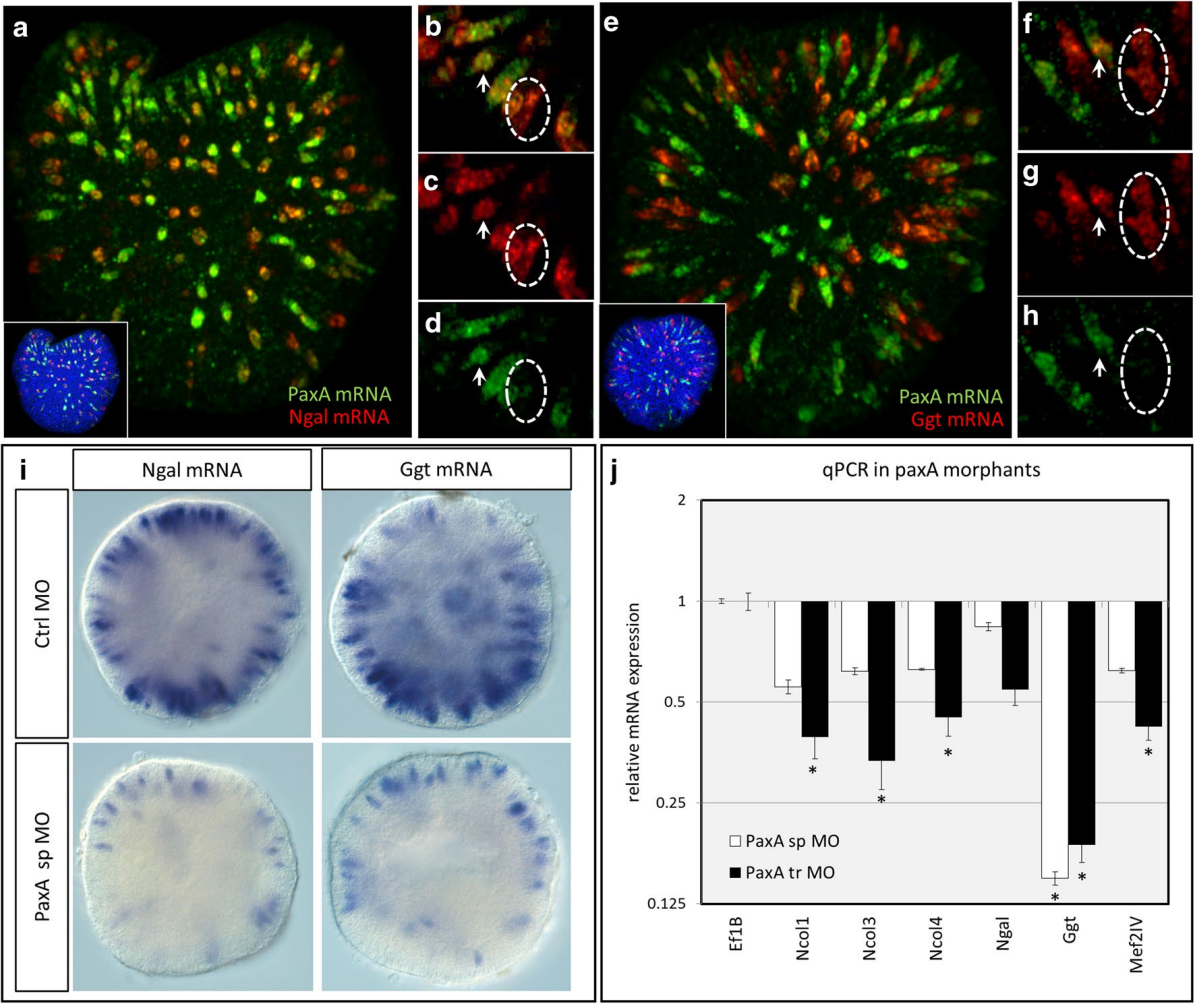
Knockdown of *PaxA* results in downregulation of cnidocyte-specific genes. **a**–**h** *PaxA* is largely co-expressed with **a**–**d** *Ngal* and **e**–**h** *Ggt*. *Arrows* indicate cells expressing both *markers*, and *dotted circles* indicate cells expressing *Ngal* or *Ggt* only. **i** Knockdown of *PaxA* results in a decrease in the number of cells expressing *Ngal* and *Ggt* (relative to control morpholino) and **j** downregulation of other cnidocyte-specific genes, relative to embryos injected with control MO. *Asterisk* indicates significant difference relative to *Ef1B* expression. Main images **a**, **e** are 3D renderings of confocal z-stacks; high-magnification images are single optical sections. Images in **i** are DIC micrographs

Finally, we examined the effect of *PaxA* knockdown on two newly identified markers of differentiated cnidocytes in *N. vectensis*—*Ngal* and *Ggt* (Fig. 10). We first demonstrate that *PaxA* is co-expressed with *Ngal* (Fig. 10a–d) in developing cnidocytes, but some cells seem to express *Ngal* without *PaxA* (Fig. 10b–d). Similarly, *PaxA* and *Ggt* are also largely co-expressed (Fig. 10e–h) though cells expressing *Ggt* only can also be detected (Fig. 10f–h). Without antibodies directed against the proteins encoded by these genes, it is not possible to determine whether this variation is spatial or temporal. Knockdown of *PaxA* by splice-blocking morpholino reduces the number of cells expressing *Ngal* and *Ggt* (Fig. 10i) and results in decreased expression (via qRT-PCR) of *Ncol1*, *Ncol3*, *Ncol4*, *Ngal*, *Ggt*, and *Mef2IV* (Fig. 10j) relative to embryos injected with control morpholino. These results confirm that *PaxA* is required for wild-type expression of all cnidocyte markers identified thus far and suggest the cells expressing *Ngal* or *Ggt* in the absence of PaxA may reflect temporal differences in expression.

**Fig. 11:**
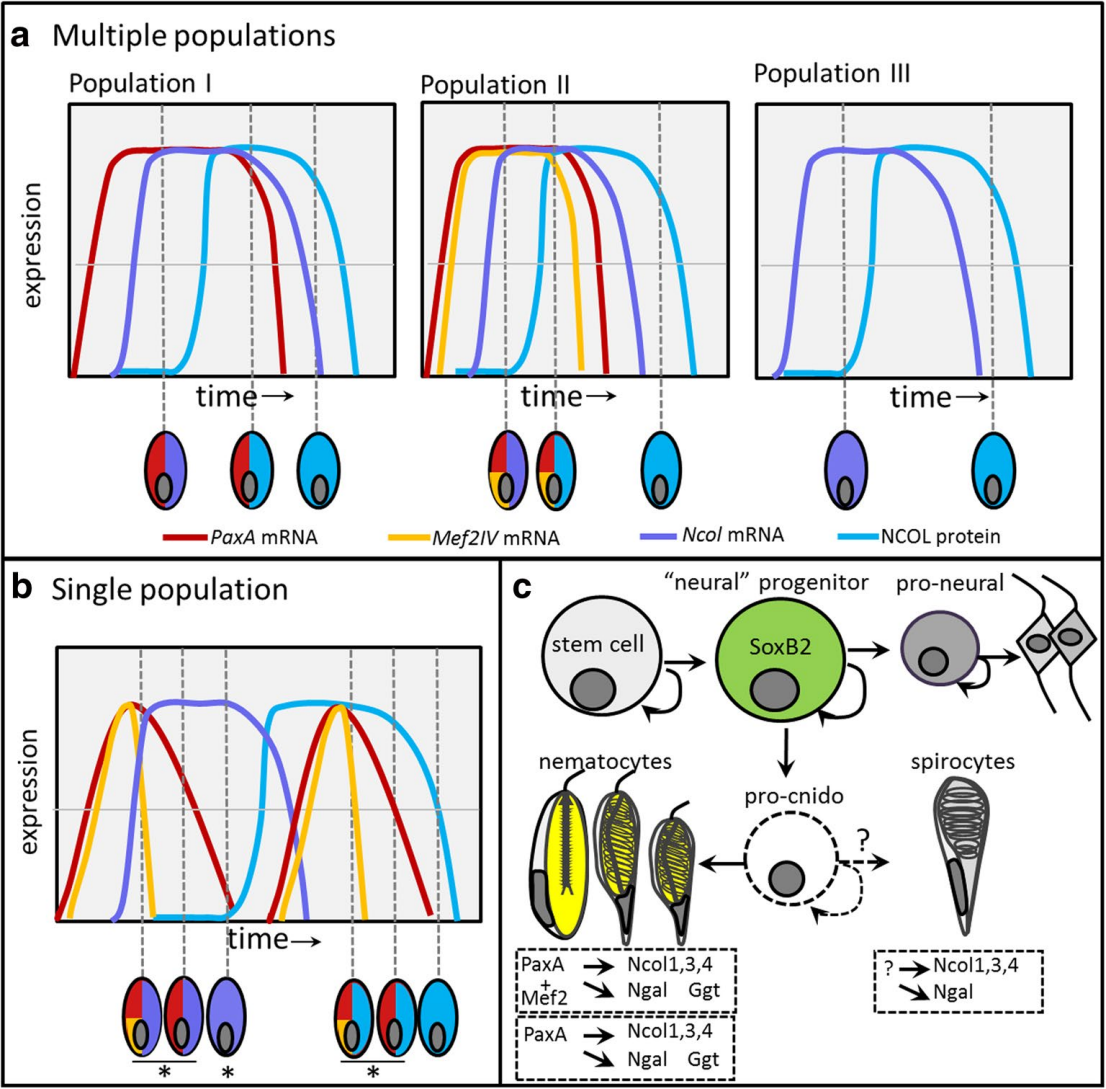
Cnidogenesis in *N. vectensis* is temporally and spatially dynamic. **a** Hypothesis 1: Cnidocytes develop from multiple independent populations of cells all present in the same epithelium at the same time. **b** Hypothesis 2: There is a single population of developing cnidocytes and all combinations of gene expression presented here result from temporal shifts in gene expression. In both cases, *Ncol* mRNA (*purple*) must be expressed before NCOL protein is detectable (*blue*) and, when expressed, *PaxA* (*red*) and *Mef2IV* (*yellow*) must be upregulated before *Ncol* mRNA. *Horizontal lines* in **a**, **b** represent an arbitrary threshold of detectable expression. **c** *SoxB2*-expressing progenitor cells give rise to both neurons and cnidocytes in *N. vectensis*. Post-mitotic neurons differentiate from cells expressing pro-neural genes, though the existence of “pro-cnido” genes (expressed in a progenitor that gives rise only to cnidocytes) is still only hypothesized (indicated by *dotted lines*). Three populations of cnidocytes may be present. Two populations differ only in the expression of *Mef2IV* and are hypothesized to become either basitrichous isorhizas and microbasic p-mastigophores or the two distinct size classes of basitrichous isorhizas. A third population expresses *Ncol1*, *Ncol3*, *Ncol4*, and *Ngal*, but the transcription factor that activates expression of these cnidocyte-specific genes is unknown. We hypothesize that this population of cnidocytes becomes spirocytes

Our combined gene and protein expression data suggest that cnidogenesis in *N. vectensis* is both temporally and spatially dynamic (Fig. 11). The results of our temporal analysis of Ncol expression (summarized in Fig. 2u) and our spatial analysis of *PaxA*/*Mef2IV* co-expression (Fig. 8) suggest there are two hypotheses that are consistent with our observations. First, the developing cnidocytes we observed may comprise multiple distinct cell populations, differentiated by their unique molecular profiles. We detected cells in the gastrula-stage embryo co-expressing *PaxA* and *Ncol*, *PaxA*, *Mef2IV*, and *Ncol*, and cells expressing *Ncol* without *PaxA* or *Mef2IV* (Fig. 8a–j). We also detected late-stage cnidocytes (Fig. 2u, stage IV) expressing *PaxA* or *PaxA* and *Mef2IV* with NCOL protein (Fig. 8k–r). The simplest explanation for these results is that one population of cells upregulates *PaxA* before *Ncol* and that *PaxA* expression continues to be detectable through late capsule development (Fig. 11a, Population I), while a second population upregulates *PaxA* followed by *Mef2IV*, both of which remain detectable through late capsule development (Fig. 11a, Population II). The observation of *Ncol*-expressing cells that lack *PaxA* and *Mef2IV* expression suggests the presence of a third population of cells for which the transcription factor(s) that upregulate *Ncol* have yet to be identified (Fig. 11a, Population III). Alternatively, the developing cnidocytes examined in this study may comprise a single, temporally dynamic population of cells, such that the various combinations of gene expression reported here mark different stages in the lifespan of a single developing cell (Fig. 11b). Two important observations make this hypothesis less plausible than the previous. First, we observed cells in the gastrula-stage embryo co-expressing *PaxA*/*Mef2IV*/*Ncol* and cells expressing *PaxA*/*Ncol* only. Because *PaxA* and *Mef2IV* must be upregulated before *Ncol*, these results can only be explained by a downregulation of *Mef2IV* before downregulation of *PaxA* during early cnidogenesis (before stage IV of capsule development). Second, we detected late-stage cnidocytes expressing *PaxA* alone and those co-expressing *PaxA*/*Mef2IV*. The only way to attribute these observations to a single temporally dynamic population of cells is to infer that both *PaxA* and *Mef2IV* are expressed bimodally with two peaks of expression in early and late in cnidogenesis. Although this is possible, the presence of multiple cell populations seems a simpler explanation. At present, all cnidocytes are thought to arise by differentiation from a *SoxB2*-expressing progenitor cell that also gives rise to neurons (Fig. 11c). A committed population of cnidocyte-specific progenitor cells has not yet been identified, but the incomplete loss of cnidocytes (Fig. 6) and incomplete knockdown of cnidocyte-specific genes in response to *SoxB2* and *PaxA* knockdowns (Figs. 6, 9, 10) suggest either that some cnidocytes develop independent of *SoxB2*, or that there are additional permissive steps in this pathway that remain to be characterized.

## Discussion

*Pax* genes are known to play a role in the development of diverse sensory structures, but this study is the first to demonstrate a role for this conserved family of transcription factors in the development of a phylum-specific cell type. We show that *PaxA*, but not *PaxC*, is expressed in a population of developing cnidocytes that also express *Ncol3* (Fig. 8) and that knockdown of *PaxA* results in loss of cnidocytes (Figs. 9, 10). Further, we demonstrate that knockdown of the neural progenitor cell-specific transcription factor *SoxB2* results in loss of *PaxA*-expressing cells (Fig. 7). Together, these results suggest that *PaxA*-expressing cells differentiate from *SoxB2*-expressing cells to give rise to early-stage cnidocytes. *PaxA* has been shown to be expressed in tissues where cnidogenesis occurs in both hydrozoans [42, 51] and scyphozoans [47, 52], yet the potential role of *PaxA* in regulating cnidocyte development in other cnidarians has not been studied. Because they differentiate from the same population of progenitor cells that give rise to neurons, cnidocytes are considered by many to be “neural” cell types; perhaps then these data support an ancestral role for *Pax* genes in metazoan neural specification. Interestingly, *PaxA* and *PaxC* seem to have arisen from a cnidarian-specific duplication of the ancestral gene that gave rise to pox-neuro in bilaterians [44, 47]. If the ancestral function of this gene was neural specification, perhaps the duplication event that gave rise to *PaxA* and *PaxC* enabled the neofunctionalization of one of these paralogs for a novel role in patterning cnidocytes in the stem cnidarian. If true, *PaxA* may well play a role in the specification of cnidocytes across cnidarians. A recent study of the hydrozoan *Hydractinia echinata* demonstrated that *SoxB2* is required for cnidogenesis [29]. Characterizing the shared and divergent parts of the cnidogenesis pathways in multiple divergent lineages of cnidarians would be a powerful way to identify the mechanisms that generate morphological and functional diversity in this cell lineage.

Besides *PaxA*, the function of the other *Pax* genes in *N. vectensis* remains largely unknown. The expression of *PaxC* in isolated ectodermal cells in two different species of anthozoans (*N. vectensis* and *Acropora digitifera*) led to early hypotheses that this gene may also be involved in neurogenesis [44, 46, 53]. The morphology of the *PaxC*-expressing cells in *N. vectensis* is consistent with the morphology of neurons (Figs. 7, 8); however, our functional experiments suggest that *PaxC* is not under the control of *SoxB2* as *PaxC* expression did not change following knockdown of *SoxB2* (Fig. 7). If these *PaxC*-expressing cells really are neurons, then *SoxB2*-expressing cells must comprise only a subset of the complete population of “neural” progenitor cells in *N. vectensis*. Finally, knockdown of *PaxC* resulted in a minor increase in the number of cnidocytes in the gastrula-stage embryo (Fig. 9), suggesting that the abundance of these two cell types (*PaxC*-expressing cells and developing cnidocytes) may be linked by some kind of negative feedback interaction. These observations are intriguing, and understanding the relationships among *SoxB2*, *PaxC*, and developing cnidocytes is likely to reveal a cryptic level of cell diversity in *N. vectensis*.

A previous study of *Mef2* function in *N. vectensis* demonstrated both a causal relationship between *Mef2IV* and *Ncol3* expression in the planula stage and an earlier role (pre-gastrula) for maternally expressed *Mef2* splice variants in patterning the endoderm [31]. In this study, we did not observe any *Mef2IV*-expressing cells that did not also express *PaxA* and *Ncol* and none of the *Mef2IV*-expressing cells were found in the endoderm (Fig. 8). These results are consistent with a cnidocyte-specific role for *Mef2IV* in *N. vectensis* as has been suggested previously [50] and further demonstrate that *Mef2IV* regulates the development of only a subset of developing cnidocytes (Fig. 11). In the coral *A. millepora*, *Mef2* is expressed in the blastopore during gastrulation, where it is thought to play a role in endoderm specification [54]; thus, both within and across species, the role of *Mef2* in patterning cnidocytes seems to be variable. Like *Pax* genes, orthologs of *Mef2* are present in the genomes of ctenophores [55], sponges [36], and placozoans (NCBI accession: XP_002110582.1), but the function of this transcription factor in non-bilaterian metazoans is largely undescribed. If *Mef2* were shown to play a role in regulating the development of the muscle cells in ctenophores, this could support the hypothesized homology of muscle cells across metazoa. Independent of its ancestral function, *Mef2* appears to have been co-opted to play a role in cnidocyte specification in *N. vectensis*. Unlike *Pax* genes, however, *Mef2* functional diversification appears to be the result of alternative splicing rather than duplication/divergence, as this gene is present in only a single copy across non-bilaterian genomes.

Quantitative investigation of the co-expression of *PaxA*, *Mef2IV*, and *Ncol3* suggests a cryptic level of cnidocyte diversity in *N. vectensis*. Zenkert et al. [22] reported that over 90% of the cnidocytes present in planula-stage larvae are basitrichous isorhizas. In this study, many, but not all, of the *Ncol3*-expressing cells also expressed *PaxA* (Fig. 8d–f) and approximately half of the cells expressing *PaxA* also expressed *Mef2IV* (Fig. 8g–j). These data are consistent with three distinct molecular fingerprints for different lineages of developing cnidocytes in *N. vectensis* (Fig. 11a) and suggest there may be variation that has gone unnoticed in previous studies of cnidocyte development in this species. Careful examination of the mature cnidocytes from larvae may in fact reveal morphological variation that correlates with the molecular variation we describe here.

Beyond the role of *Pax* genes in cnidocyte specification, we have demonstrated that the cell dynamics of cnidogenesis in *N. vectensis* differs in many ways from *Hydra* and other hydrozoan model systems. Using markers of different stages of cnidogenesis, we demonstrate that cnidocytes develop individually and asynchronously in the ectoderm of *N. vectensis* in all life stages (Figs. 2, 3). It is, therefore, common to find early- and late-stage cnidocytes adjacent to each other in the same epithelium at the same time in *N. vectensis*. This is in contrast with the mode of cnidocyte development in hydrozoans, whereby groups of cnidocytes differentiate synchronously from a centralized population of progenitor cells before migrating to the tissue in which they will be deployed [56]. As such, cnidocyte development is highly position dependent in *Hydra* with progressive stages of developing cnidocytes occurring at regular and predictable distances from the progenitor cell population [57, 58]. This is not the case in *N. vectensis* where progenitor cells are scattered throughout the epithelium in which new cnidocytes are differentiating individually [27]. Furthermore, we have shown that *Ncol1*, *Ncol3*, and *Ncol4* are all co-expressed in all cnidocytes in *N. vectensis* (Fig. 4), rather than being spatially restricted as previously reported. Our data suggest that the differences in minicollagen expression across cnidocyte types may be temporal, as differences in mRNA expression are not corroborated by differences in protein expression. While transcription begins at approximately the same time (as evidenced by dFISH), the timing of translation appears to vary slightly. In particular, it appears that NCOL3 may undergo translation slightly earlier than NCOL4 or NCOL1 and that the latter two may continue to be detectable by α-NCOL antibodies even after NCOL3 is not.

Minicollagens are known to be essential for cnidocyte development across cnidarians, but several recent studies suggest there are additional features that may be shared across cnidocytes. Indeed, chondroitin sulfate has been shown to play a role in scaffolding minicollagens during capsule construction in both *Hydra* and *N. vectensis* [59]. Furthermore, *Ngal* is known to be expressed in cnidocysts from hydrozoans [6, 20], cubozoans [15], anthozoans (this study), and even the polar capsules of myxozoans [8]. Thus, *Ncol*, *Ngal*, and chondroitin sulfate together seem to comprise a minimal kernel of genes necessary for synthesis of the cnidocyst across cnidarians. *Ggt* was identified as one of the nematocyte-specific genes in *Hydra* [20] and was reported to be expressed in the ectodermal tissue of the tentacles and body wall of the sea anemone *M. senile* [60]. This enzyme appears to have evolved before the origin of cnidocytes as many orthologs are present outside of eukaryotes [61], and therefore may have been co-opted to play a role in generating the extreme osmotic pressure that enables nematocysts to fire with such force [12]. Interestingly, the matrix of spirocytes does not generate the same intracapsular pressure as nematocysts and, likewise, does not react with DAPI, suggesting *Ggt* may not be (as) active during development of spirocytes. It is possible, then, that the lineage of cnidocytes we identified as expressing *Ncol3* and *Ngal* but not *Ggt* could be developing spirocytes. Alternatively, a study of poly-γ-glutamate in the sea anemone *M. senile* suggested that the levels of this amino acid are highest in microbasic b-mastigophores in this species [62]. Given that *Ggt* expression was not detected in the body wall of tentacle bud stage animals but was detected in the tentacles (Fig. 3), an alternative hypothesis is that the cells expressing *Ncol3* and *Ngal* without *Ggt* may be the small size class of basitrichs in *N. vectensis*. Chemical inhibition of γ-glutamyl transpeptidase activity and/or knockout of the *Ggt* gene in vivo would therefore be expected to result in animals with only spirocytes and small basitrichs. This experiment would be a valuable next step in understanding the generation of cnidocyte subtypes.

## Conclusions

Expression of minicollagen is the unifying molecular feature of cnidocytes, and cnidocyte diversity has been suggested to result from differences in the spatiotemporal expression of various minicollagen paralogs during cnidogenesis [1, 63]. Interestingly, all minicollagens appear to be co-expressed in *N. vectensis*, but only a subset of cells express minicollagen under the control of *PaxA*. Indeed, we show that two conserved families of transcription factors are co-expressed in only a subset of developing cnidocytes, suggesting the combined expression of many conserved genes may be sufficient to generate diversity in a novel cell lineage. This system serves as a valuable model for exploring the relationships between the secreted or structural proteins that impart cell identity (e.g., minicollagen and nematogalectin) and the transcription factors responsible for initiating expression of these genes. After the origin of the ancestral cnidocyte, diversity in this cell lineage may have been generated as different classes of conserved transcription factors were recruited to activate the expression of novel structural genes. Examining the diversity of transcription factor families known to activate expression of cnidocyte structural genes across cnidarians will be an important contribution to understanding how diversity evolved in this novel cell lineage.

## Methods

### Animal care and tissue collection

Adult sea anemones were maintained in the dark at 17 °C and spawned as described previously [64]. In vitro fertilization was performed at room temperature (25 °C), and fertilized egg masses were de-jellied by agitation in 1/3X filtered seawater (FSW) containing 4% l-cysteine (C-7352, Sigma, USA). Liberated zygotes were washed three times in clean 1/3X FSW to remove excess cysteine and reared at 17 °C or 25 °C in the dark. At the time of collection, embryos were immobilized in 7% MgCl_2_ for up to 10 min at 25 °C, fixed in 4% paraformaldehyde (PFA) with 0.2% glutaraldehyde for 1 min at 25 °C, and then fixed in 4% PFA without glutaraldehyde for 1 h at 4 °C. For in situ hybridization, fixed tissues were washed three times in phosphate-buffered saline containing 0.1% Tween 20 (PTw), washed once in sterile 0.2-μm-filtered autoclaved deionized water (sH_2_O), washed once in 100% methanol, and transferred to clean 100% methanol for storage at −20 °C. For immunohistochemistry, tissues were fixed in a similar manner, washed three times in PTw, and stored at 4 °C in clean PTw before processing. Embryos subjected to ISH followed by IHC analysis were fixed as described for ISH embryos. Staging of embryos is indicated for development at two different temperatures (Table 2).

**Table 2:** Timing of collection for embryos of the indicated stages

	Blastula (h)	Gastrula (h)	Early planula (h)	Late planula (h)	Tentacle bud (h)	Primary polyp (h)
@ 16 °C	24	48	72–96	144–168	240–264	336^+^
@ 25 °C	12	24	48–60	72–84	96–120	168^+^

Time is presented as hours (h) post-fertilization

### Cloning and probe synthesis

To generate a wild-type cDNA library, we extracted total RNA from embryos at multiple stages of development: blastula, gastrula, early and late planula, tentacle bud, and primary polyp stages. Pooled embryos were homogenized in TRI reagent (T9424, Sigma, USA) with a mechanical pestle for 30 s at 25 °C, and total mRNA was extracted following the manufacturer’s instructions for TRI reagent. cDNA synthesis was performed following the manufacturer’s instructions for the Advantage RT-for-PCR kit (639506, Clontech, USA), and samples were stored at −20 °C until use. To generate RNA probes for in situ hybridization, target sequences (approximately 1000 bp each) were amplified from our cDNA library using a standard PCR protocol, separated by gel electrophoresis on a 1% agarose gel, isolated using the QiaQuick gel extraction kit (28704, Qiagen, USA), and cloned into the PGEM-T vector (A3600, Promega, USA) following the manufacturer’s protocols. Targets were PCR-amplified from plasmids using standard SP6 and T7 primers, gel-purified (as above), and used as template for in vitro transcription using the Megascript SP6 or T7 transcription kit (AM1330/AM1334, Ambion, USA). Purified mRNA was solubilized in nuclease-free water and then diluted to a stock concentration of 100 ng/μl in hybridization buffer for storage at −20 °C. At the time of use, probes were further diluted to a working concentration of 1 ng/μl in hybridization buffer and heated to 90 °C for 5 min before application to tissues.

### In situ hybridization and immunohistochemistry

In situ hybridization (ISH) was performed as previously described for single-color ISH [65] and for double-fluorescent ISH [66]. Immunohistochemistry (IHC) assays were performed as described previously [64] using *Nematostella vectensis*-specific antibodies directed against minicollagens 1, 3, and 4 (α-NCOL1, α-NCOL3, and α-NCOL4) which were originally designed and validated by Zenkert et al. [22]. Nuclei were counter-stained for 30 min at 25 °C with 1.43 μM DAPI, and mature cnidocytes were labeled by incubation in 143 μM DAPI (in PTw with 10 mM EDTA) for 30 min at 25 °C. Confocal imaging was performed on a Zeiss LSM 710 microscope, and assessments of cnidocyte abundance were performed in 3D reconstructed images produced from confocal z-stacks. Counting was automated using Imaris software (Bitplane, Concord, MA, USA), and the proportion of embryonic cells comprised of cnidocytes was calculated as the ratio of cells labeled with antibody to nuclei labeled with DAPI. Differences among treatments were evaluated using ANOVA in the R statistical computing environment [67].

### Manipulation of gene expression

Splice- and translation-blocking morpholinos were purchased from Gene Tools, LLC (Philomath, OR, USA), reconstituted in nuclease-free water to a final concentration of 1 mM, and stored as directed by the manufacturer at 25 °C in the dark before use. Just prior to microinjection, morpholinos were heated to 60 °C for 5 min and centrifuged for 1 min before being diluted to a final working concentration with 0.2 mg/ml Alexa Flour-555 conjugated dextran (Invitrogen, USA) in nuclease-free water. Morpholinos were microinjected into uncleaved zygotes following the protocol of [68], and embryos were reared to the time of collection at 25 °C in the dark. For splice-blocking morpholinos, disruption of proper splicing was confirmed using PCR and gel electrophoresis with cDNA synthesized from morphant embryos and primers designed to amplify the sequence targeted by the morpholino (see Fig. 9; Table 3 for primer sequences). To assay the effects of ectopic mRNA expression, the complete *PaxA* coding sequence was cloned upstream of the fluorescent reporter Venus and purified RNA was transcribed from this construct using the mMessage mMachine kit (AM1348, Ambion, USA) following the protocols of Roure et al. [69] and Rottinger et al. [70]. To confirm the specificity of the *PaxA*::*Venus* construct in rescuing the morpholino phenotypes, we also injected SoxB2 ATG MO and PaxA sp MO in combination with a publicly available human *histone H2B*::*GFP* construct (NCBI AB591038) [71]. Both constructs were microinjected alone, or mixed with morpholino before being injected into fertilized eggs, and embryos were assessed visually for translation of reporter proteins before being reared for additional assays. The effects of in vivo gene manipulation on the expression of putative target genes were assayed using ISH/IHC (above) or by qRT-PCR.

**Table 3:** Primers used in this study

Target gene	Use	Forward (5′ → 3′)	Reverse (5′ → 3′)
*Ncol1*	ISH	ATGGCGTTCAAGATCACACTGT	CTATTTGCAACACGCTGGGGC
*Ncol1*	qRT-PCR	TGGCGTTCAAGATCACACTGTTGTG	GCATGGATTAGCGTCACGTTTTTGC
*Ncol3*	ISH	ATGGCGTCGAAACTCATCCTCGGAGT	TTATCTTCTGCCAGCGCAGCATCCCG
*Ncol3*	qRT-PCR	AGATGGCGTCGAAACTCATCCTCG	TTGGCGCTGCGTTTGTATGTGC
*Ncol4*	ISH	ATGAGGAATAGAGTTCTCCTACTGCT	CTATTTGCCGCGACTGCAGCA
*Ncol4*	qRT-PCR	TCTGAAGAGAAGTCCCAACCCATGC	TGAACGAGCAACAAGCGGAAGC
*Ngal*	ISH	GGGGCAGTTAGGAGGCTACAGA	ACCGCTTCTCCTTGGCTGCT
*Ngal*	qRT-PCR	AAGCAGTACGAGCTCATCAAGG	CTGGCACTGTCCTTTGACCTTC
*Ggt*	ISH	CGGCATTGTGTGTAGGCGTGGT	AGGGCTTGCGGTGAGGAAGGAA
*Ggt*	qRT-PCR	ATACTCGTTCACCCAGTGGTTC	CATCTGTCTGTTGTGGATGCTG
*Mef2IV*	ISH	CACCATGGGGCGAAAGAAGATCCAG	CACATAGCTTTGCTCAGATATAG
*Mef2IV*	qRT-PCR	CGCATTAGCAAGACAAAGCAAGTGC	TGCAAAAAGCATTCCCCGTACTGAC
*PaxA*	ISH	ATGCCTCATAGAGGTCCT	GCCCTGGGTCAGTGTATCAAGG
*PaxA*	qRT-PCR	CAGGGAAGTCACCAATGGAGA	GCCTTTAACACTGACCAACTCG
*PaxA*	sp MO	ATGCCTCATAGAGGTCCT	GCCCTGGGTCAGTGTATCAAGG
*PaxC*	ISH	ATGGCTCACCAAATTCCATTCCAC	AACCGGAAGTCGATTGTGTCTC
*PaxC*	qRT-PCR	CAAGTCTCCTGGGTACGATCAC	TATCGTGCTTCGCTCTTCTGTG
*PaxC*	sp MO	ATGGCTCACCAAATTCCATTCCAC	AACCGGAAGTCGATTGTGTCTC
*SoxB2*	ISH	CGAGGCAAGAGAAAGCATTACG	ACACTCGTTGGTTACTCAGCTC
*Elav* ^a^	qRT-PCR	GCGGTCTACCGAAAGACATGAA	CTACCTTCGCCGCTCACATC
*RFamide* ^a^	qRT-PCR	TCGGTCGCACAATGGATACC	TCCTTAGCAGCTTGTCGCTT
*Ef1B* ^a^	qRT-PCR	TGCTGCATCAGAACAGAAACCTGC	TAAGCCTTCAAGCGTTCTTGCCTG

^a^Primers sequences originally presented by Richards and Rentzsch [30]

### Quantitative real-time PCR (qRT-PCR)

We performed qRT-PCR as described previously [64]. In short, qRT-PCR was performed in morphant cDNA samples collected from multiple independent microinjection experiments per morpholino (*N* = 5 for control morpholino, *N* = 2 for PaxA tr MO, and *N* = 3 for all other morpholinos). Data are reported as mean expression values of target genes in target morpholino-injected embryos relative to mean expression values in control morpholino-injected embryos. The expression of elongation factor 1β (*Ef1B*) in target and control embryos is set to 1.0, and all other expression values are reported relative to this on a log_2_ scale. Differences in relative mRNA expression among target genes were assessed using ANOVA in R [67].

## Abbreviations

cDNA: complimentary DNA
DAPI: 4′,6-diamidino-2-phenylindole
FSW: filtered seawater
ISH: in situ hybridization
IHC: immunohistochemistry
MO: morpholino
mRNA: messenger RNA
PCR: polymerase chain reaction
PFA: paraformaldehyde
PTw: phosphate-buffered saline with 0.1% Tween 20
qRT-PCR: quantitative real-time PCR
